# The metal face of protein tyrosine phosphatase 1B^☆^

**DOI:** 10.1016/j.ccr.2016.07.002

**Published:** 2016-11-15

**Authors:** Elisa Bellomo, Kshetrimayum Birla Singh, Alberto Massarotti, Christer Hogstrand, Wolfgang Maret

**Affiliations:** a Metal Metabolism Group, Division of Diabetes and Nutritional Sciences, Faculty of Life Sciences and Medicine, King’s College London, London, UK; b Dipartimento di Scienze del Farmaco, Università del Piemonte Orientale “A. Avogadro”, Novara, Italy

## Abstract

A new paradigm in metallobiochemistry describes the activation of inactive metalloenzymes by metal ion removal. Protein tyrosine phosphatases (PTPs) do not seem to require a metal ion for enzymatic activity. However, both metal cations and metal anions modulate their enzymatic activity. One binding site is the phosphate binding site at the catalytic cysteine residue. Oxyanions with structural similarity to phosphate, such as vanadate, inhibit the enzyme with nanomolar to micromolar affinities. In addition, zinc ions (Zn^2+^) inhibit with picomolar to nanomolar affinities. We mapped the cation binding site close to the anion binding site and established a specific mechanism of inhibition occurring only in the closed conformation of the enzyme when the catalytic cysteine is phosphorylated and the catalytic aspartate moves into the active site. We discuss this dual inhibition by anions and cations here for PTP1B, the most thoroughly investigated protein tyrosine phosphatase. The significance of the inhibition in phosphorylation signaling is becoming apparent only from the functions of PTP1B in the biological context of metal cations as cellular signaling ions. Zinc ion signals complement redox signals but provide a different type of control and longer lasting inhibition on a biological time scale owing to the specificity and affinity of zinc ions for coordination environments. Inhibitor design for PTP1B and other PTPs is a major area of research activity and interest owing to their prominent roles in metabolic regulation in health and disease, in particular cancer and diabetes. Our results explain the apparent dichotomy of both cations (Zn^2+^) and oxyanions such as vanadate inhibiting PTP1B and having insulin-enhancing (“anti-diabetic”) effects and suggest different approaches, namely targeting PTPs in the cell by affecting their physiological modulators and considering a metallodrug approach that builds on the knowledge of the insulin-enhancing effects of both zinc and vanadium compounds.

## Highlights

• Modulation of phosphatase activity by metal cations and metal oxyanions.
• Zinc inhibition of protein tyrosine phosphatase 1B.
• Structural chemistry of inhibitory metal sites in proteins.
• Metal buffering in enzymatic assays.

## 1 Introduction

Over sixty years ago, the late Bert L. Vallee suggested that “[metal ions] may accelerate or inhibit reactions selectively. With reference to the latter, one can point out that about 50 percent of all known enzymes require free sulfhydryl groups for activity: it is well known that metal ions can react with sulfhydryl groups, thereby inhibiting these enzymes. It is completely unknown whether the body avails itself for inhibiting, activating, or both actions of metal – or neither – to control enzyme reactions” [1]. Even today, it remains largely unknown to which extent the transition metal ions and zinc *control* enzymatic reactions. Clearly, metal ions activate apoenzymes to form active holoenzymes, *i.e.* metalloenzymes, but this aspect of metallobiochemistry is not considered part of regulation. It is estimated that about 50% of all enzymes require a metal ion, and it is assumed that metalloenzymes are 100% metallated and that the metal ion remains bound during the lifetime of the protein. However, a paradigm for the opposite situation is emerging, namely an active apoenzyme without a metal ion and an inactive holoenzyme with a bound metal ion [2]. This is borne out by the observation of different types of enzymes with diads or triads of potentially metal ion-coordinating catalytic groups in the active site being very tightly inhibited by zinc ions [3], [4]. This inhibition suggests metallation states different from 100% occupancy, and modulation of enzyme activities by fluctuating metal ion concentrations. In this article, we will discuss protein tyrosine phosphatases (PTPs) as metal-modulated enzymes. In contrast to other phosphatases, many of which are active metalloenzymes, PTPs are generally not recognized as metalloenzymes but bind metal cations such as Zn^2+^ and metal anions such as vanadate extremely tightly and with concomitant inhibition. The fact that zinc ion concentrations in the cell are in the range where such enzyme inhibition is observed strongly supports the idea that zinc(II) modulates protein tyrosine phosphatase activity *in vivo* [2]. Physiological metal ion modulation is different from metal ion inhibition of PTPs as a mechanism of toxicity or exploration of metal ligand complexes for pharmacological regulation.

Zinc(II) is now known to be a cellular signaling ion, but targets have not been confirmed. PTPs are likely targets as phosphorylation states of many signaling proteins depend on changes in cellular zinc(II). It appears that we have missed a major area of metabolic regulation, in particular a role of zinc ions in the most significant way of signal transduction, namely phosphorylation signaling, and that the number of enzymes that interact with zinc(II) is significantly larger than the already impressive estimate that about 3000 human proteins may require zinc(II) as a cofactor [5].

### 1.1 Protein tyrosine phosphatases (PTPs)

Protein tyrosine phosphorylation is a dynamic process that regulates many cellular processes through the activities of kinases and phosphatases. Dysregulation of either one of these enzymes is linked to multiple diseases. The human genome contains 107 PTPs divided into several subgroups [6]. The cysteine-based PTPs constitute the largest group, which includes the dual-specificity and classical tyrosine-only transmembrane, receptor-like PTPs (RT-PTP) and non-transmembrane PTPs [7]. Among the classical non-transmembrane PTPs is PTP1B (PTPN1), with a catalytic N-terminal domain, a regulatory region, and a membrane-localization domain that tethers the enzyme to the cytosolic side of the endoplasmic reticulum (ER) [8].

PTP1B dephosphorylates several substrates including the insulin and leptin receptors, hence having an important role in metabolism. PTP1B knock-out (KO) mice are more sensitive to insulin and have lower serum glucose and insulin levels; when put on a high fat diet, they are resistant to weight gain and insulin. Moreover, the phosphorylation of the insulin receptor (IR) in both skeletal muscle and liver is increased in KO compared to control mice [9]. Transgenic mice overexpressing PTP1B specifically in the muscles show impaired insulin sensitivity [10]. Although mutations of the PTP1B gene have not been linked to cancer and despite the fact that PTP1B regulates the activity of both the epidermal growth factor (EGF) and the platelet-derived growth factor (PDGF) receptors [11], the enzyme is considered a tumor suppressor. Overexpression of PTP1B in fibroblasts protects the cells from transformation [12], and increased PTP1B expression is observed in breast and ovarian cancer patients, possibly in response to increased levels of tyrosine phosphorylation [13], [14]. However, the actual role or modulation of PTP1B in cancer treatment or prevention is still under debate. Owing to these critical cellular functions, PTP1B has been the subject of numerous investigations.

PTP activity is tightly regulated by a plethora of mechanisms including oxidation, phosphorylation, sumoylation, and dimerization [15], [16], [17], [18], [19]. In recent years, evidence accumulated that metal ions may also regulate PTP activity. Here we review metal ion regulation of PTPs and address its coordination chemistry and significance, with a focus on PTP1B.

### 1.2 The PTP1B-catalyzed reaction

**Fig. 1:**
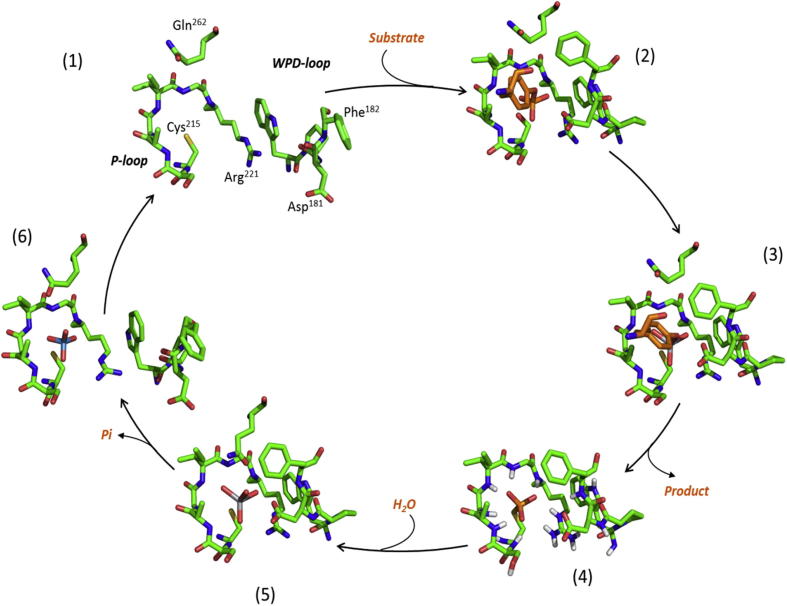
Protein tyrosine phosphatases catalyze the dephosphorylation of the substrate by a ping-pong mechanism. The enzyme initially is in a resting state with the WPD loop open (PDB id: 2CM2). Upon substrate (in orange) entry into the catalytic pocket, the WPD loop closes (PDB id: 1PTU, Michaelis complex). The enzyme goes through the first transition state (PDB id: 3I7Z) and the first product is released with the enzyme left in a phospho-cysteine intermediate (PDB id: 1A5Y, Q262A mutant). A water molecule is subsequently activated in the second transition state (PDB id: 3I80), before the WPD loop opens again (PDB id: 2HNQ, Michaelis complex) and the inorganic phosphate is released. Modified from Brandao et al., [24]; reproduced with permission.

PTP1B is the prototype of protein tyrosine phosphatases, originally isolated from placenta [20]. The catalytic domain of PTP1B consists of 280 residues. The enzymatic mechanism responsible for the dephosphorylation of phosphorylated substrates involves engagement of the phospho-tyrosine substrate to PTP1B and promotion of a conformational change of the so-called WPD loop that moves closer to the phospho-tyrosine and allows the side chain of Asp^181^ to act as a general acid/base. The side chain of Arg^221^ changes orientation and assists the closure of the WPD loop. Interactions between the re-positioned Arg^221^, which stabilizes the phosphate group, and the Trp^179^ and Pro^180^ residues stabilize the WDP loop in the closed conformation. The sulfhydryl group of Cys^215^ is the catalytic residue. It has a characteristically low p*K*_a_ of 5.5 [21] and performs a nucleophilic attack on the phosphate group of the phospho-tyrosine substrate while Asp^181^ transfers a proton to the tyrosine oxygen group of the leaving substrate. In the second step, the resulting phospho-cysteine intermediate is hydrolyzed. Gln^262^ aligns a water molecule for the nucleophilic attack on the phospho-cysteine intermediate while Asp^181^ now functions as a general base accepting a proton (Fig. 1). Asp^181^ is thought to have a very high p*K*_a_ of 6.8 [22]; it moves 8 Å when the WPD loop closes and is thought to be protonated in the closed conformation [22]. The removal of the phosphate from the catalytic cysteine leads to the re-opening of the WPD loop, terminating the catalytic cycle [23], [24].

### 1.3 Measuring enzymatic activity in the presence of metal ions

PTP activity is measured with several enzymatic assays; most of them involve the use of a small molecule or a peptide. Dephosphorylation of these substrates can be detected by fluorescence (*e.g.*, 6,8-Difluoro-4-Methylumbelliferyl Phosphate, DiFMUP), electronic absorption (*e.g.*, *p*-nitrophenyl phosphate, pNPP) or radioactivity (*e.g.*, ^32^P-labeled phospho-peptides) [25]. Many published experiments are based on an end-point assay. Quenching the reaction at a given time point and calculating the amount of product formed does not provide evidence for linear rates and thus important characteristics of the enzymes are missed. Therefore, determining the activity under steady-state conditions with initial rates is superior for mechanistic investigations. If metal ion binding is competitive with substrate binding, then IC_50_ values relative to the substrate concentration employed in the assays are obtained. The true *K*_i_ values can be significantly lower. For example, an IC_50_ value of 98 pM was estimated for receptor protein tyrosine phosphatase β (PTPRB), but the *K*_i_ value is actually 21 pM after a full kinetic analysis was performed [26].

When measuring the activity of enzymes in the presence of metal ions, their concentrations are rarely controlled. If inhibition by metal ions occurs at very low concentrations, spurious amounts of metal ions are already present as contaminants in control reactions in the absence of any added metal ions, even when the chemicals in all solutions are certified by commercial metal analysis. Hence, additional metal analyses and the use of a metal ion buffer to control metal ion concentrations may be necessary for analyzing inhibition. Another issue is that typical assay conditions for PTPs involve additional agents that participate in metal ion buffering; their metal ion binding capacity must be considered. The origin of the protein is also important. Most PTPs used in research are recombinant: they are produced by individual investigators or bought from companies that focus on the preparation of active enzymes. The proteins usually contain EDTA, glutathione (GSH), dithiothreitol (DTT), or other metal ion chelating agents that compete with the protein for metal ion binding. The presence of these agents will influence the free metal ion concentrations. Therefore, when assays are performed in the absence of a metal ion buffer, the reported inhibition constants may need to be re-evaluated as the inhibition is likely to be stronger.

In our experiments, we employ metal ion buffers that control the free metal ion concentrations. The choice of the chelating agent as a metal ion buffer depends on the desired buffering range, and for the measurement of PTPs it is nitrilotriacetic acid (NTA), as it buffers metal ions such as Zn(II), Cd(II) and Cu(II) in the range where inhibition is observed. Moreover, we measure the total amount of metal present in the solutions by ICP-MS. Using software such as MaxChelator [27] or MINEQL+ 4.6 (Environmental Research Software, Hallowell, ME), we then calculate the free metal ion concentration from the overall composition of the buffer, including the concentration of metal ion chelators present in the solution of the protein, and the measured and added concentrations of metal ions [28]. In this way we obtain the free metal ion concentrations for each of the total metal ion concentrations added (Table 1). The differences found when comparing the output from the two software packages is probably due to the different algorithms used and the fact that, while MaxChelator calculates free metal ion concentrations using only the total metal ion and chelator concentrations, MINEQL+ also takes into account other buffer components. The free metal ion concentrations are significantly different from the total metal ion concentrations. In fact, this metal ion buffering *in vitro* is not any different from the situation *in vivo*, where all metal ions are buffered in specific ranges by proteins and small molecules.

## 2 Metal ion coordination in PTP1B

Metal ions bind to proteins through interaction with the donors of amino acid side chains, the ligands. The cellular concentrations of the different metal ions vary substantially, and for some metal ions participating in cell signaling, the concentrations fluctuate and depend on the status of the cell.

The coordination number is governed by the properties of both the metal ions (*e.g.*, charges, size) and the ligands (*e.g.*, electronegativity). Coordination numbers have been analyzed by employing the Cambridge Structural Database [29]. Although most metal ions have preferences towards a particular coordination number, they can assume different geometries and have more than one coordination number. Moreover, metal ions have ligand preferences (Table 2).

Although the tabulation shows that arginine and glutamine side chains are not typical ligands for zinc ions and other metal ions, there are at least 20 established cases where proteins coordinate zinc ions with glutamine [32].

### 2.1 Metal ion binding to PTP1B

The enzymatic activity of PTPs is affected by both metal cations and (metal) oxyanions. Among those, zinc ions (Zn^2+^) and vanadate ions (VO_4_^3−^) were originally described as inhibitors of PTP1B [20].

### 2.2 Metal cations inhibit PTPs

We interrogated the Protein Data Bank (PDB) for metal cations (Cd(II), Cu(II), Zn(II), Fe(II)) bound to human PTP1B. None of the 129 crystal structures deposited contains any of these metal ions near the catalytic site. This finding, however, does not mean that metal ions are unable to bind to PTP1B.

#### 2.2.1 Zinc(II)

Zinc(II) has been known as an inhibitor of PTPs since the early 1980s [33]. Since then, others confirmed inhibition and reported increasingly higher IC_50_ or *K*_i_ values [28], [34]. What is often overlooked, however, is that, compared to other metal ions such as cadmium(II) or copper(II), the inhibition of PTPs by zinc(II) occurs at biologically relevant metal ion concentrations. PTP1B is not the only protein tyrosine phosphatase inhibited by zinc ions. Other PTPs are also inhibited (Table 3).

Zinc ions also inhibit PTPs in cultured cells. When glioma cells were incubated with zinc ion concentrations in the presence of the zinc(II) ionophore pyrithione, a time-dependent increase in protein tyrosine phosphorylation was observed [34], [35]. Zinc ions also activate the epidermal growth factor receptor (EGFR) in human bronchial epithelial cells. Although the activation fails to trigger the dimerization of EGFR typically induced by EGF, it increases phosphorylation, which is accompanied by a loss of PTP activity. In particular, zinc ions caused an inhibition of PTP1B, suggesting that the mechanism responsible for zinc ion-induced activation of EGFR signaling is dependent on the inhibition of PTP1B by Zn^2+^ [36], [37], [38].

When we examined the inhibition of PTP1B by zinc ions, we observed that the enzyme remained at least 50% active even in the presence of high zinc ion concentrations. This observation is not unique to PTP1B. Both PTPRG and HePTP behave in similar ways (M. Wilson, MSc thesis, King’s College London). Because the dephosphorylated substrate is released in the first catalytic step, the retention of 50% activity suggests that the enzyme is inhibited in the second catalytic step when it is still in the phosphorylated form. This observation would indeed explain the failure to see complete inhibition even at high zinc ion concentrations [28]. We have recently investigated the possible binding sites of zinc(II) to PTP1B with molecular docking [28]. Since the enzyme undergoes conformational changes during the catalytic cycle, we investigated zinc(II) binding to three different conformations of the protein: the enzyme in the open conformation (PDB id: 2CM2), the enzyme in the closed conformation with the phospho-cysteine intermediate (PDB id: 1A5Y) and the dephosphorylated enzyme in the closed conformation, which was obtained by removing vanadate from the second transition state structure (PDB id: 3I80). For each of the structures, we found several zinc ion-binding sites at the periphery of the enzyme (Table 4, [28]).

**Fig. 2:**
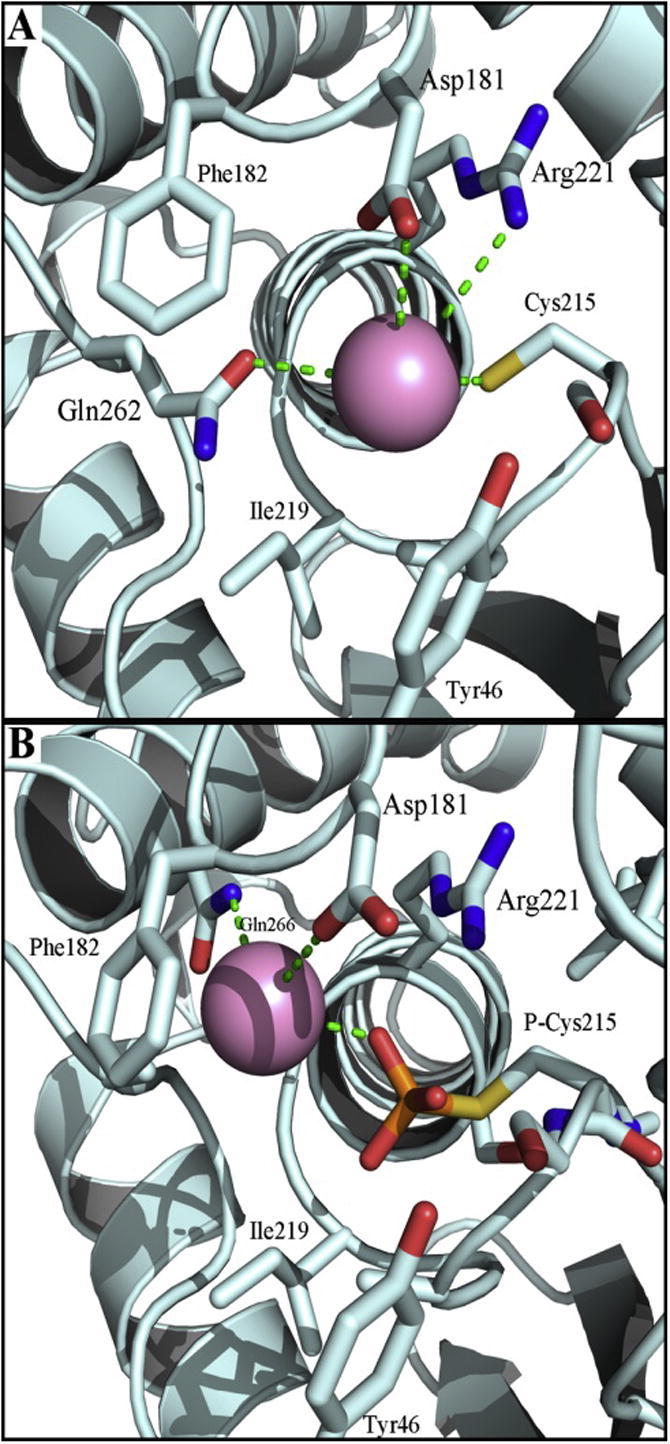
Docking simulation performed on different PTP1B crystal structures. (A) Closed form (PDB id: 3I80) and (B) phospho-cysteine intermediate (PDP id: 1A5Y). Since the position of the zinc ion in the two structures differs by about 4 Å Gln266 in (A) is too far for coordination. Gln^262^ is not shown in (B) as the structure was obtained on a Gln262Ala mutant to avoid hydrolysis of the phospho-cysteine intermediate. Protein structure is represented in pale cyan, zinc ion as pink sphere, and relevant amino acids as sticks [28].

Remarkably, zinc ion binding to the catalytic site was observed only when the conformational change of the WPD loop has occurred and the catalytic residue Asp^181^ has moved into the active site (PDB id: 1A5Y, pose 3 and PDB id: 3I80, pose 4). In the dephosphorylated form of the enzyme (obtained by removing vanadate from the structure), zinc(II) is in a coordination sphere surrounded by Asp^181^, Arg^221^, Gln^262^ and Cys^215^ (Fig.2A). In the phospho-intermediate form, phospho-Cys^215^, Asp^181^ and Gln^266^ and Gln^262^ are all in the inner coordination sphere of zinc(II) (Fig.2B). The crystal structure of this form examined has a Gln262Ala mutation, hence Gln^262^ coordination of zinc(II) is not shown in Fig.2B but it has a critical position for coordination (Fig.2A). If Gln^262^ is re-introduced in the position *in silico*, the coordination of the docked zinc ion is very strong with a distance of about 1.5 Å. If Tyr^46^ changes its position, it may become a ligand as well. The open conformation fails to accommodate zinc(II) in the catalytic pocket. The idea that zinc(II) binds to the catalytic site is supported by the observations that zinc(II) is a competitive inhibitor of PTPRB [26]. When comparing the two binding modes (Fig.2A and B), the center of the zinc ion moves at least 4 Å. The inhibitory effect of zinc(II) is expected to be twofold. When Gln^262^ and Asp^181^ become ligands of zinc(II) in the closed conformation, Gln^262^ no longer aligns a water molecule for catalysis and Asp^181^ can no longer serve as the catalytic base for the dephosphorylation of the phospho-cysteine intermediate in the second step of the reaction. Hence zinc(II) traps the enzyme in the phospho-cysteine form. The coordination of zinc(II) in this form is exclusively from oxygen donors.

Though the zinc(II) site in the closed conformation of PTP1B is unlike typical zinc(II) coordination in zinc metalloenzymes, it is not without precedence. The number of oxygen donors is sufficient to provide a strong binding site as zinc(II) binds to the magnesium(II) site in phosphoglucomutase and inhibits the enzyme with picomolar affinity [40]. In PTP1B, Gln^266^ (1 oxygen), Asp^181^ (1–2), phosphate (1–2), and Gln^262^ (1) could all contribute oxygen ligands. Gln has been identified as a ligand of zinc(II) in proteins [32]. Alternatively, or additionally, water molecules may be ligands. Hydrogen bonding of metal ion-bound water molecules also makes an energetic contribution to the stability of metal ion sites in proteins. The implication of zinc(II) locking the enzyme in the phosphorylated state is that the site is now no longer accessible for substrate. We believe that zinc(II) binding in the active site in the closed conformation in the absence of phosphate (Fig.2A) is not relevant as the closed conformation would require the prior presence of substrate, *e.g.* the phosphorylated insulin receptor. Zinc(II) would not be an insulin-mimetic as the insulin receptor would not function in signaling if PTP were to remain bound to it. We cannot rule out, however, that zinc(II) locks the enzyme in a closed conformation once the second substrate, *i.e.* the phosphate group, has left the catalytic site. At present, there does not seem to be a precedent for this mechanism as the cleavage of the phospho-cysteine intermediate is coupled to the transition of the enzyme from the closed to the open conformation.

#### 2.2.2 Iron(II)

Mononuclear di-citrate (cit) iron complexes interact with PTPs and inhibit their activities [41]. Specifically, while Fe:cit prepared with Fe(III) in 1:1 and 1:2 ratios did not inhibit tyrosine phosphatases, inhibition was observed for the 1:4 and the 1:10 Fe:cit preparations (at concentrations between 250 and 500 μM of the complex) [41]. Fe^2+^ inactivates recombinant PTPs (PTP1B, CD45, and LAR) in a concentration-dependent manner [42]. Since reactive oxygen species are implicated in regulating PTP1B activity, and iron(II) is involved in the Fenton reaction (decomposing H_2_O_2_), the interaction of H_2_O_2_ and Fe^2+^ with PTP1B was also examined. Physiological concentrations of Fe^2+^ (∼500 nM, [43]) not only inactivated the enzyme less than H_2_O_2_ but also prevented its inactivation by H_2_O_2_, possibly by generating hydroxyl radicals which, although oxidizing, are unspecific reactants likely unable to reach the catalytic site of PTPs because they would not diffuse that far *in vivo*. Fe^2+^ also inhibits the dual specificity protein phosphatase VHR and the inhibition was reversible by addition of EDTA, suggesting a direct binding of iron(II) to this PTP [44].

#### 2.2.3 Copper(II)

Inhibition of PTPs has been explored with copper(II) complexes. The inhibition constants vary between low nM to low μM values [45], [46], [47], [48]. The effect of the free metal ion, though unbuffered, was also examined for both PTP1B and the dual specificity protein phosphatase VHR. Copper(II) strongly inhibited with IC_50_ values of 100 nM and 1 μM for VHR and PTP1B, respectively. However, enzymatic activity was not fully recovered by adding EDTA. Only the presence of reducing agents reactivated the enzymes completely, suggesting that copper(II) oxidizes the catalytic cysteine [44]. Considering the very low free cytosolic copper ion concentrations and the fact that intracellular copper is mostly in the reduced state (copper(I)), these experiments are not physiologically significant. When investigating PTP1B in the presence of increasing concentrations of Cu^2+^ and a metal buffering system, we found a much lower IC_50_ value of 0.3 pM (unpublished data, K. Birla Singh).

#### 2.2.4 Cadmium(II)

Although Cd^2+^ inhibits other phosphatases, there is no report on its effect on PTPs [49]. When PTP1B was investigated in the presence of increasing concentrations of Cd^2+^ in an NTA-containing buffer, an apparent inhibition constant of ≈5 nM was obtained (unpublished data, K. Birla Singh), very similar to the one for zinc(II) (5.6 nM), based on concentrations of free cadmium ions calculated as indicated above for zinc.

### 2.3 Oxyanions inhibit PTP activity

Inorganic phosphate is the hydrolytic product of the PTP reaction. It is a competitive inhibitor of PTP1B with a *K*_i_ value of ∼17 mM [50]. Other oxyanions with structural similarities to the phosphate group inhibit PTPs with different affinities: molybdate, arsenate [50], tungstate and vanadate. When analyzing the inhibition of PTP1B under our experimental conditions, apparent *K*_i_ (IC_50_) values of 1.5 μM (vanadate), 9 μM (heptamolybdate), 210 μM (tungstate), 200 μM (arsenate), and 54 mM (nitrate) were measured (unpublished data, K. Birla Singh). The different inhibition constants may reflect the different ionization states of the anion at the pH of investigation, the propensity of vanadate to form a covalent intermediate analogous to the phospho-intermediate in contrast to the other anions, which form Michaelis-like complexes, and geometric factors, such as in the case of nitrate, which has a planar geometry (Table 5).

#### 2.3.1 Vanadate(V) and oxidovanadium(IV)

**Fig. 3:**
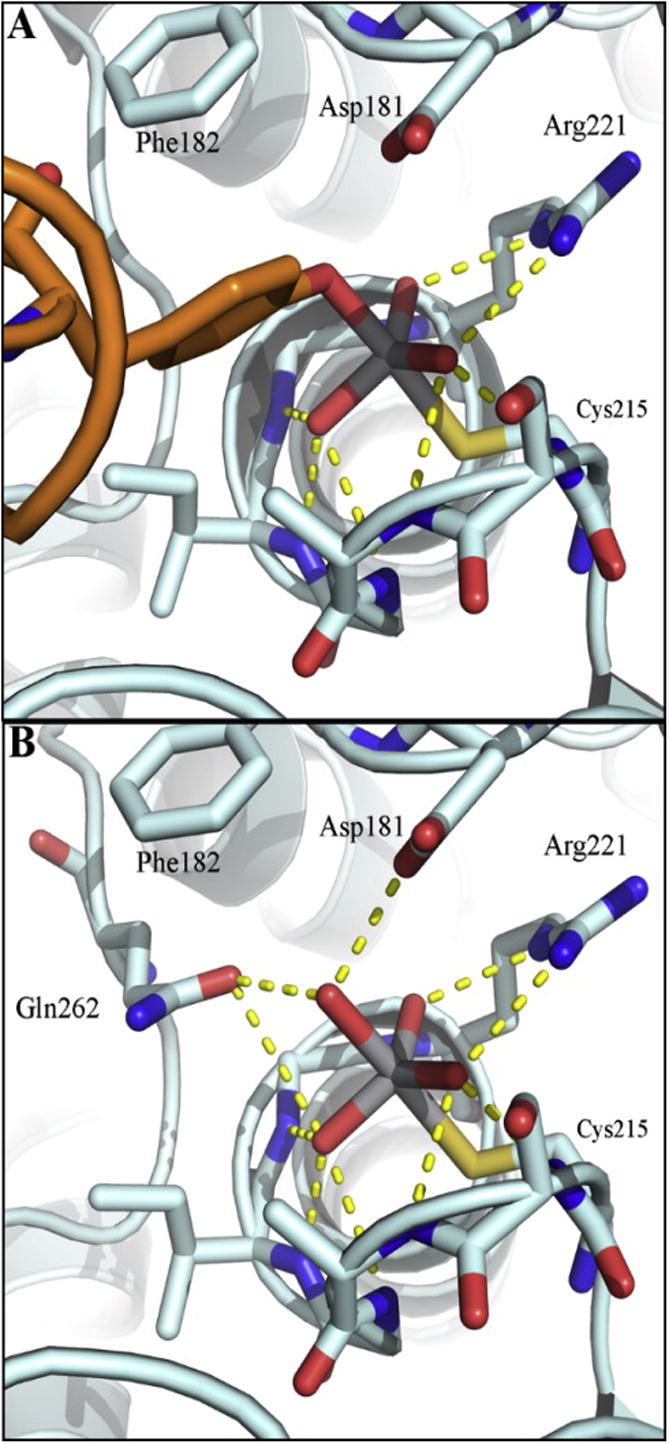
X-ray crystal structure of (A) first transition state complex between PTP1B, metavanadate, and the Tyr in the peptide DADEYL (PDB id: 3I7Z) and (B) second transition state complex between PTP1B and orthovanadate (PDB id: 3I80) [24]. Protein structure is represented in pale cyan, peptide DADEYL in orange, and vanadate and relevant amino acids as sticks. H-bonds are depicted as yellow dot lines.

The bioinorganic coordination chemistry of vanadium is discussed in a number of very recent reviews, covering vanadate in structural biology [51], the reaction dynamics of vanadium ions [52], binding, transport, and storage of vanadium in cells [53], vanadium in medicine [54], [55], and vanadium as inhibitors of phosphatases [56]. Vanadate has structural similarities with phosphate, and was therefore suggested to be a transition state analog of phosphate in phosphatases [57]. However, the two anions differ significantly in their acid/base and redox chemistries. The p*K*_a2_ values are 7.8 for dihydrogen vanadate (H_2_VO_4_^−^) and 7.2 for dihydrogen phosphate. Thus there is comparatively less di-anion (HVO_4_^2^^−^) at pH 7.0. Only the oxidation state +5 of the non-metal phosphorous is relevant for mammalian biology, whereas minimally two oxidation states of the metal vanadium, +4 and +5, are important. Thus phosphorous is redox-inert whereas vanadium is redox-active in biology. Vanadate was discovered as a strong inhibitor of (Na/K)-ATPase in a commercial ATP preparation (*K*_i_ = 40 nM in the presence of 25 mM Mg^2+^) [58]. It is a competitive inhibitor of PTP1B with a *K*_i_ of 0.38 ± 0.02 μM (pH 7.3) [59]. In contrast to other oxyanions such as sulfate, vanadate, like phosphate, forms a covalent intermediate with the enzyme. Vanadate inhibits bovine low molecular weight PTP competitively with a *K*_i_ of 1.0 ± 0.6 μM (pH 7.5) and forms a trigonal bipyramidal (tbp) S_N_2 transition state [60]. While a square planar geometry of the 5-coordinate complexes of vanadate is preferred in solution, the geometry of intermediates is tbp in a large number of phosphatases [56], [61]. Analysis of geometry was based on angular parameters using *τ*-values, the ratio of the angle of the axial vs the equatorial ligands in tbp geometry divided by 60, *i.e.*, *τ* = (*β* − *α*)/60, which, for an ideal geometry with *β* = 180° and *α* = 60°, gives a *τ*-value of 1 [62]. In the covalent intermediate of PTP1B, the sulfur donor of the catalytic cysteine becomes the fifth ligand of vanadium(V) with a rather long bond distance (2.4–2.5 Å in the two transition states) and deviations from the ideal tbp transition state of vanadium(V) with *τ*-values ranging from 0.83 (PDB id: 3I7Z) to 0.90 (PDB id: 3I80) and structures described best as umbrella-like inversions with deviations of ligands from the equatorial plane in exploded transition states, a term referring to increased bond lengths of the breaking and forming bonds. Further investigations employing shape analysis – continuous symmetry measures [63] – demonstrated that the umbrella-like distortions of the tpb species are on a pathway from and to the tetrahedral anions [64]. These characteristics make vanadate a transition state analog for PTPs [65]. Vanadate and other oxyanions that form product complexes like phosphate have been used for 3D-structural elucidation of the enzymatic mechanism of PTP1B. The structures of two transition states of PTP1B with vanadate ions were analyzed crystallographically [24]. The structures share close structural characteristics with the metaphosphate and orthophosphate transition states in phosphate transfer reactions [65]. The first transition state was obtained by crystallizing PTP1B in the presence of metavanadate and a peptide, while the second transition state was obtained by incubating PTP1B crystals with the peptide and orthovanadate. In both transition states, vanadate binds to the sulfur of Cys^215^; the equatorial oxygen atoms bind to the amide groups of the P-loop (Ser^216^, Ala^217^, Gly^218^ and Ile^219^) and to the guanidinium group of Arg^221^. Gln^262^ in the first transition state is rotated compared to the position in the second transition state, where it binds the apical oxygen in the trigonal vanadate moiety [24] (Fig.3A and B). The motion of Gln^262^ is critically important as it aligns a water molecule as a nucleophile for releasing the second product, phosphate, from the intermediate. Together the structures of complexes with different oxyanions, *i.e.*, phosphate, vanadate, and tungstate were instrumental in formulating the reaction pathway of PTP1B (Fig. 1).

Pervanadate, which contains monoperoxo or diperoxo ligand bridges in the equatorial position, also inhibits PTPs and is a more potent insulin-mimetic than vanadate [66]. However, the mechanism of inhibition is different from vanadate. Pervanadate is an irreversible inhibitor of PTPs. Mass spectrometric analyses revealed that addition of pervanadate to the enzyme resulted in the oxidation of the catalytic cysteine to a sulfonic acid derivative [59].

**Fig. 4:**
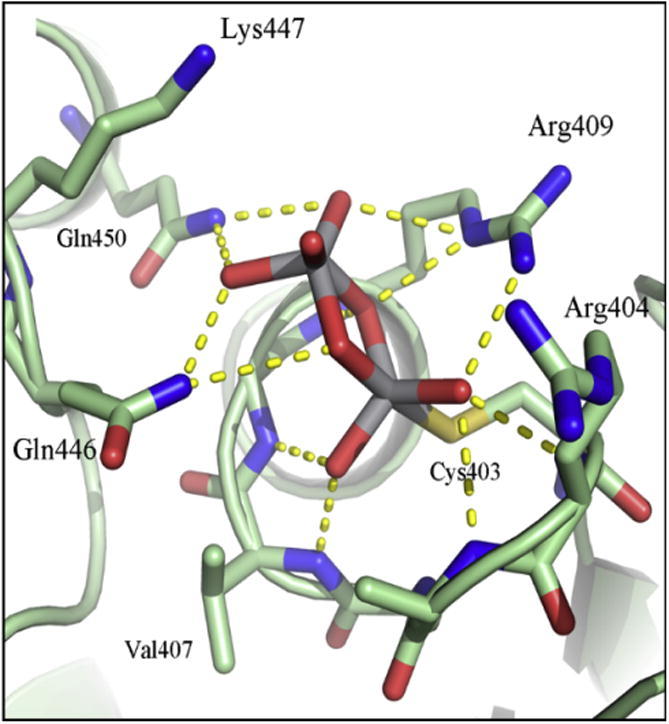
X-ray crystal structure of *Yersinia enterocolitica* PTP (R235C, W354F) complexed with divanadate (PDB id: 3F9B) [67]. Protein structure is represented in pale green, and vanadate and relevant amino acids as sticks. H-bonds are depicted as yellow dot lines.

Crystals of the *Yersinia enterocolitica* PTP YopH, in which the Trp in the WPD loop had been mutated to a Phe, grown in the presence of vanadate, showed electron density that was interpreted as a divanadate (V_2_O_7_*^x^*^−^) ion bound in the active site [67]. This particular species of vanadate has never been observed in wild type Yersinia PTP or in any other PTP. A 2.2 Å structure with vanadate is deposited in the PDB (id: 2I42) and is discussed as having a mononuclear VO_4_S covalent intermediate [56]. Although vanadate forms oligomers in solution, wild type, active PTPs are expected to catalyze the decomposition of dimeric forms in solution with the same mechanism used for phospho-peptide substrates [68]. The lengths of the V–S and V–O bonds were similar to those observed in the structures of PTP and monomeric vanadate; the second vanadium atom is coordinated in a distorted trigonal bipyramid with two bridging oxygens. The other three oxygens bind to the NH atoms of Gln^446^ and Gln^450^, to a water molecule, and to the amino group of Lys^447^ (Fig. 4). The structure with divanadate is intriguing as it demonstrates that indeed two metal ions can be accommodated in the active site of a PTP.

Vanadate undergoes multiple chemical reactions, such as complexation with many commonly employed buffers, reduction, and olation to form polyoxymetallates [69], [70], [71]. Because of this complex chemistry, it is important to determine the species interacting with proteins at physiological pH. For example, H_2_VO_4_^−^, the main species at pH 7, can increase its coordination number by binding additional water molecules or dimerize to divanadate, tetravanadate, or species of higher nuclearity, in particular at higher concentrations. In order to minimize oligomerization, it is recommended to prepare stock solutions of vanadate >0.1 mM at pH 10 and boil the solution [72]. Compounds with thiols such as glutathione and ascorbate reduce vanadium(V) to vanadium(IV) [52], [56]. The reduction was investigated in the cytoplasm of human erythrocytes after uptake of vanadate and advanced to explain the inability of exogenously added vanadate to inhibit the (Na/K)-ATPase *in vivo* [73]. The reduction of vanadate(V) to vanadium(IV) species may also be important in the presence of thiol-based reducing agents typically employed in assays of PTPs. Whether vanadate is an oxidant for the catalytic cysteine in PTP1B has not been considered and/or examined, however. The best known salt of V(IV) is vanadyl sulfate, which contains the oxidovanadium (“vanadyl”) cation [V(IV)O]^2+^. This cation is also an inhibitor of PTP1B with a *K*_i_ value of 0.11 μM (pH 7.0, 37 °C) [74]. It must be kept in mind, however, that upon dissolution of vanadyl sulfate a significant fraction of vanadium(IV) oxidizes immediately to vanadium(V) at neutral pH. Quite appropriately, it was discussed how an anion as well as a cation can be an inhibitor of phosphatases [71]. The answer lies in the speciation of the cation. The vanadyl cation is stable at acidic pH and the hydrated species [VO(H_2_O)_5_]^2+^ with a square pyramidal geometry undergoes consecutive hydrolysis when the pH increases to form anionic species at nanomolar to submicromolar concentrations: [VO(H_2_O)_4_(OH)]^−^, [(VO)_2_(OH)_5_]^−^, and [VO(OH)_3_]^−^. Thus, there are anionic and cationic species in equilibrium with insoluble {VO(OH)_2_}*_n_* and with [(VO)_2_(OH)_5_]^−^ being a prominent species at neutral pH [71]. In addition, vanadium(V) also exists in both cationic and anionic forms [75]. In acidic solutions, the cationic species is [V(V)O_2_]^+^, which binds to proteins such as transferrin [75]. Coordination of vanadium(IV) and vanadium(V) ions with biological ligands is another factor of speciation in a biological matrix [70].

In addition to inorganic vanadate(V) and oxidovanadium(IV), many organic vanadium(V) and vanadium(IV) compounds were investigated as inhibitors of PTP1B owing to their potential as antidiabetic agents and for many other therapeutic indications (see below). Vanadium complexes inhibit PTP1B with IC_50_ values between 30 nM and about 1 μM but rarely do they show pronounced selectivity for a particular phosphatase [76]. The actual mechanism of inhibition remains to be elucidated. So far, only vanadate has been investigated in 3D structures of PTPs. The rationale for the significant activity in synthesizing metallodrugs as inhibitors of PTPs is based on the expectation that membrane permeability, compartmentalization, toxicity, and selectivity for different PTPs can be achieved by the organic ligand, thus overcoming the inherent poor selectivity of vanadate.

Vanadium compounds receive a great deal of interest as antidiabetic agents and, more recently, as potential anticancer drugs [55], [69]. Because of their favorable properties (membrane permeability, stability, and potency), work focused on organic chelates of VO^2+^, oxidovanadium(IV). VO(acac)_2_ (acac = acetylacetonate) is an uncompetitive inhibitor of PTP1B when assayed with a phosphotyrosine peptide that stimulates the epidermal growth factor receptor (EGFR) [77]. Likewise, bis-(maltolato)oxovanadium(IV) (BMOV) is a reversible inhibitor of glutathione transferase-tagged PTP1B with an IC_50_ of 0.86 ± 0.02 μM. In this case, the inhibition is mixed with *K*_i_ values of 1.2 and 2.1 μM for the enzyme and the enzyme/substrate (*p*-nitrophenyl phosphate) complex, respectively [78].

#### 2.3.2 Tungstate

**Fig. 5:**
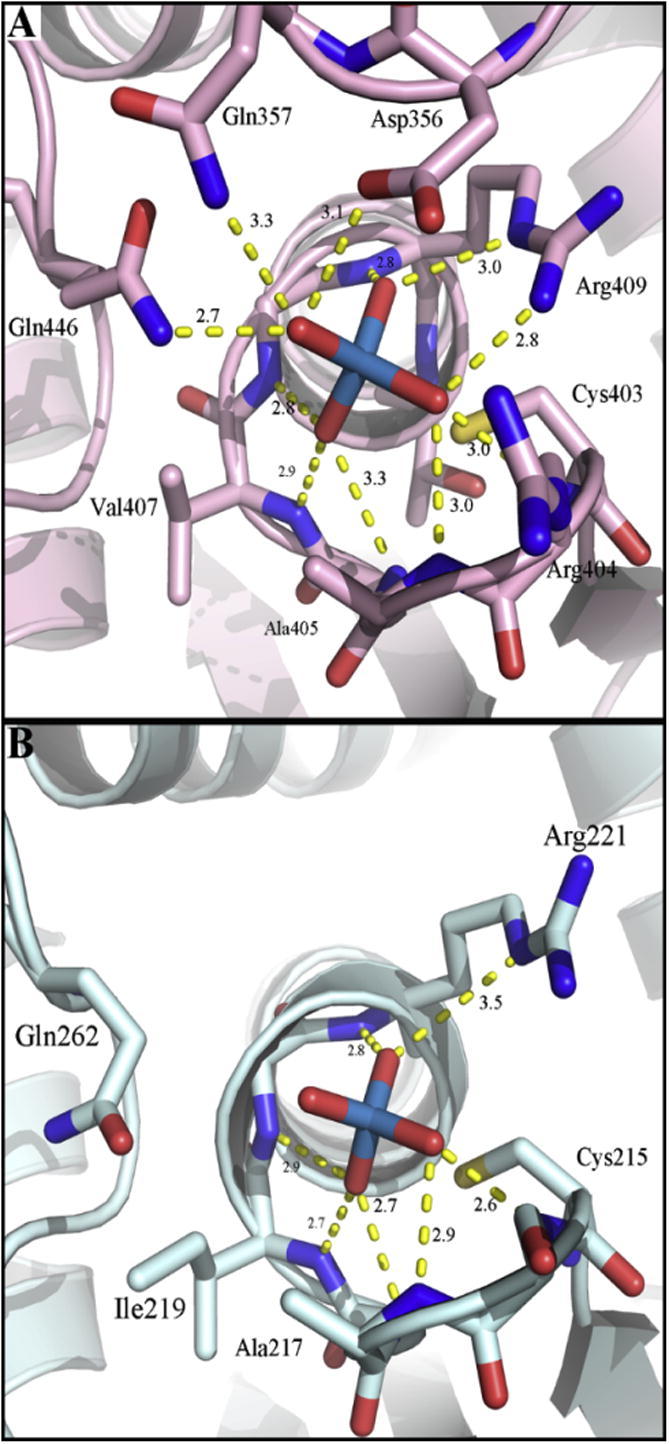
X-ray crystal structures of (A) *Yersinia* PTP (R235C) (PDB id: 1YTW) [79] and (B) human PTP1B (PDB id: 2HNQ) complexed with tungstate [80]. Protein structures are represented in pink and pale cyan respectively, tungstate and relevant amino acids as sticks. H-bonds are depicted as yellow dot lines, distances are in Å.

The crystal structure of *Yersinia* PTP (YopH) with tungstate indicates that tungstate behaves like other phosphate mimetics, namely closing the WPD loop [79] (Fig.5A). In contrast to vanadate, tungstate does not form a covalent intermediate. PTP1B has also been crystallized with tungstate [80] (Fig.5B). Two of the tungstate oxygens form hydrogen bonds with the amide groups of Ser^216^, Ile^219^ and Gly^220^. Arg^221^ also coordinates with one of the tungstate oxygens. Tungstate binding to the catalytic site of PTP1B causes conformational changes in the enzymes: the side chains of both Cys^215^ and Arg^221^ re-position towards the oxyanion. However, the WPD loop remains in the open conformation. The fact that the crystals were obtained by soaking PTP1B in the presence of high concentrations of tungstate together with the fact that the WPD loop is closed in the *Yersinia* PTP structure, suggests that binding of tungstate in the open conformation of PTP1B is an artifact of the crystallization condition.

#### 2.3.3 Molybdate

**Fig. 6:**
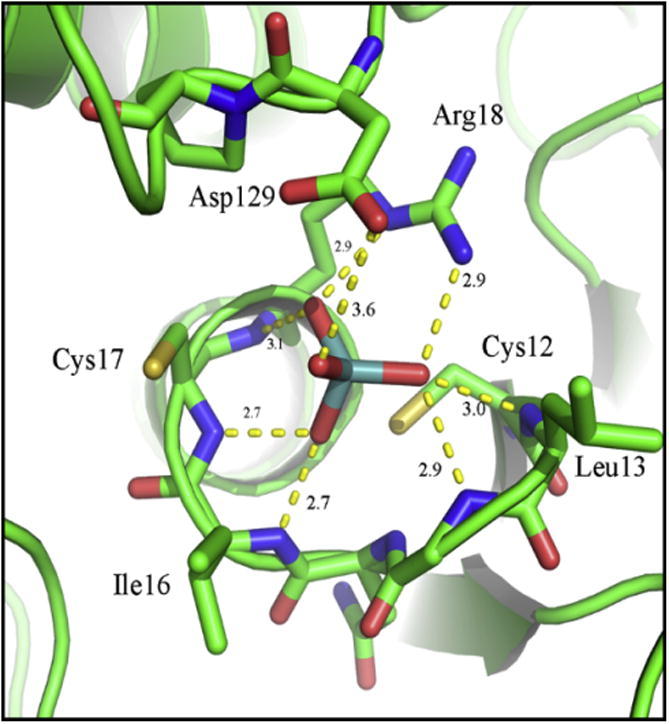
X-ray crystal structure of bovine low molecular weight PTP complexed with molybdate (PDB id: 1Z13) [60]. Protein structure is represented in green, molybdate and relevant amino acids as sticks. H-bonds are depicted as yellow dot lines, distances are in Å.

There are no available structures of PTP1B with molybdate. There is, however, a structure of the bovine low molecular weight PTP co-crystallized with molybdate [60]. In this work, the phosphatase was crystallized with tungstate, molybdate and vanadate and the inhibition constants of the metal oxyanions were measured. Vanadate was the stronger inhibitor, displaying typical tbp geometry, while molybdate and tungstate were weaker inhibitors (9 μM and 210 μM, respectively). Unlike vanadate, but similar to tungstate in PTP1B, molybdate does not form a covalent intermediate but in this case the enzyme is in the closed conformation (Fig. 6).

#### 2.3.4 Nitrate

**Fig. 7:**
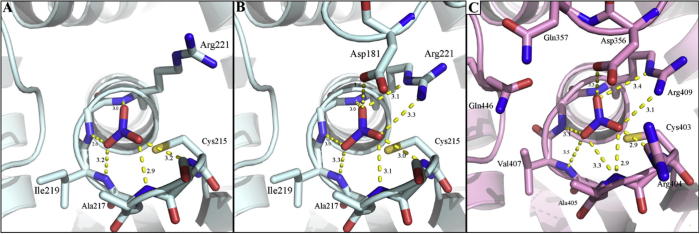
X-ray crystal structure of human PTP1B complexed with nitrate (PDB id: 4BJO): (A) open form and (B) closed form [81]. Protein structure is represented in pale cyan, and nitrate and relevant amino acids as sticks. H-bonds are depicted as yellow dot lines, distances are in Å. (C) X-ray crystal structure of *Yersinia* PTP (R235C) complexed with nitrate (PDB id: 1YTN) [79]. Protein structure is represented in pink, nitrate and relevant amino acids as sticks. H-bonds are depicted as yellow dot lines, distances are in Å.

Other oxyanions also interact with PTP1B. No information is available on the effect that nitrate (NO_3_^−^) has on PTP activity. Hence we determined its inhibition. The IC_50_ value is 54 mM (unpublished data, K. Birla Singh). Crystal structures of PTP1B in the presence of nitrate have been solved [81]. The WPD loop was captured in both an open and a closed conformation (Fig.7A and B). Nitrate is held in place by non-covalent interactions: in the closed form, it forms hydrogen bonds with Arg^221^, Asp^181^, Ser^216^, Ala^217^, and Gly^220^ (Fig.7B); in the open form, the distances to Ala^217^, Gly^220^ and Arg^221^ are slightly less and it also forms hydrogen bonds with Ile^219^ (Fig.7A). The movement of the WPD loop seems to change only slightly the position of the oxyanion in relation to the catalytic site [81]. Nitrate was also crystallized in a complex with *Yersinia* PTP [79]. In this case, the WPD loop is in the closed conformation and the coordination is virtually identical to the one observed in PTP1B (Fig.7C).

#### 2.3.5 Arsenate

Arsenate inhibits PTP1B with a *K*_i_ of 4.3 mM [50]. Under our experimental conditions the IC_50_ value is 200 μM (unpublished data, K. Birla Singh). There is no crystal structure of PTP1B with arsenate. However, arsenate reductase belongs structurally to the family of PTPs and several crystal structures have been solved [82]. Remarkably, the protein phosphatase CD45 belonging to the PTP superfamily reduces arsenate [83].

## 3 Significance of PTP1B inhibition

Both metal cations and oxyanions inhibit the activity of PTP1B. Although several metal ion complexes have been investigated with regard to inhibiting PTPs and shown promises as metallodrugs, and in the case of vanadium compounds entered clinical trials, none have developed into human therapeuticals. Given the inhibition of PTP1B by free metal ions, investigations of the interaction of metal ion complexes with PTPs need to consider the stability of the metal ion complexes in order to determine whether the inhibition is due to the metal ion complex itself or the cation dissociating from the complex. Understanding the interaction of free metal ions and/or oxyanions with PTP1B, therefore, is important in order to improve the design of potential therapeutic agents.

Investigation of vanadate as an insulin-mimetic dates back to 1899, when it was observed that oral administration of Na_3_VO_4_ to diabetic patients decreased glycosuria [84]. The study did not have any follow up until 80 years later, when it was shown that several inorganic vanadium compounds affected adipocytes similar to insulin, namely stimulating glucose transport and oxidation, and hepatocytes, namely increasing glycogen synthesis and inhibiting gluconeogenesis [85]. Notably, this work was performed at a time when the search for a molecular function of vanadium in biology was on-going, a scientific journey that started when very high (hundreds of millimolar) concentrations of vanadium were found in tunicates (ascidians) [86]. While vanadium is now well established as an essential element in nitrogenases and haloperoxidases in some branches of life, an essential molecular function in humans remains unknown. Inhibition of PTP1B by vanadate provides one explanation of the insulin-like effects of vanadate, often referred to as “insulin-mimetic” but more appropriately described as insulin-enhancing or insulin sparing, though other PTPs involved in the insulin signal transduction pathways may contribute as well [87]. Numerous studies have explored the various insulin-enhancing effects of vanadate and vanadium compounds both *in vitro* and *in vivo*. Of interest was the discovery that Na_3_VO_4_ normalized hyperglycemia in an animal model of diabetes [88], suggesting that vanadate or vanadium compounds could potentially be used for the treatment of diabetes [89]. Speciation again is an issue as vanadate and vanadium(V) compounds are thought to be reduced to vanadium(IV) compounds in cells and will form complexes with other metabolites. Moreover, in the presence of reactive species such as under conditions of peroxide signaling, there is the possibility of oxidation of vanadium(IV) to vanadium(V), *i.e.* vanadium undergoing 1-electron redox cycling, thus accounting for the formation of organic radicals and the toxicity of vanadium compounds. Among vanadium compounds, bis(ethylmaltolato)oxidovanadium(IV) (BEOV), showed encouraging results and was in clinical trials as a prodrug for the treatment of type 2 diabetes [90]. This vanadyl (VO^2+^, oxidovanadium(IV)) chelate and the corresponding acetylacetonate have been investigated in detail structurally and with regard to their synergism with insulin in cells [91]. Phase I and phase II pre-clinical trials were performed with organic vanadium compounds as antidiabetic drugs (while vanadyl sulfate and vanadate, owing to the fact that their toxicity was known, went directly into phase II clinical trials) but had to be discontinued because of renal complications [90], [92], [93]. So far, vanadium compounds join the failures of finding specific drugs that target PTP1B, which after enormous efforts were deemed “undruggable” by the pharmaceutical industry. However, PTP1B has received additional attention because it is a validated therapeutic target in Her2-positive breast cancer and inhibitors are discussed as a therapeutic approach to Rett syndrome and anxiety disorders [94], [95].

Similar to vanadate, tungstate alters glucose metabolism when added to rat hepatocytes [96]. Follow-up experiments confirmed that administration of tungstate, like vanadate, to diabetic rats normalized glycaemia and hepatic glucose metabolism [97]. Sodium tungstate has been proposed as a potential therapeutic agent for type 2 diabetes and although its effects as an inhibitor of PTP1B have been acknowledged, the antidiabetic properties of sodium tungstate may be related to effects other than that of being a PTP1B inhibitor [98]. Similar conclusions were reached when the role of tungstate as an antiplatelet agent was investigated and the effects were thought to be due to inhibition of PTP1B – at least in part [99].

Molybdate has also been widely used as a PTP inhibitor [100], [101], [102]. In contrast to vanadium and tungsten, molybdenum is an essential element for humans. Its function as a cofactor in enzymes is linked to its binding to the pterin prosthetic group. In rat hepatocytes, molybdate inhibits glycogen synthase and increases fructose bisphosphate, while it increases glucose uptake in rat adipocytes [103]. The action is likely due to an increased phosphorylation of the insulin receptor and the insulin receptor substrate [104]. Experiments with molybdenum supplementation have also been performed on diabetic rats. Molybdate decreased hyperglycemia and glycosuria, improved glucose tolerance, and replenished glycogen stores [105].

It is unclear whether the concentrations at which vanadate and other oxyanions inhibit the activity of PTP1B occur under physiological conditions. Relatively low metal oxyanions concentrations (*e.g.* vanadate, ∼1 μM) inhibit PTPs. Vanadium concentrations in human blood are 0.2–15 nM (median 50 ng/L in 108 human subjects) [106]. Based on about 1 mg of vanadium in a 70 kg human and assuming a random distribution, one can calculate an average vanadium concentration of 0.3 μM [54]. Other (non-metal) oxyanions, such as phosphate, nitrate, borate, sulfate inhibit in the millimolar range, but none of the inhibition of these ions appears to be physiologically relevant in living cells. For example, phosphate inhibits with a *K*_i_ of 17 mM while the cellular phosphate concentrations are in the range of 0.5–5 mM.

Zinc ions, on the other hand, are physiologically important inhibitors of enzymes [2]. Other metal ions, either essential metal ions such as iron(II,III) and copper(I,II) or non-essential metal ions have pathophysiological/toxicological effects on PTPs. In order to interpret metal ion inhibition of PTPs, an understanding of the cellular concentrations of total and free metal ions is necessary.

### 3.1 Physiological metal cation concentrations

Apart from the properties of the metal cation and its affinity for ligands, the actual concentration of the metal ions available for protein binding is a crucial factor in determining whether or not a protein indeed binds a particular metal ion in the cell. The factor determining the availability is the range in which metal ions are buffered in the cell. Cellular metal homeostasis is a well-controlled process, involving many transporter proteins as well as storage in organelles.

In general, the affinity of a divalent metal ion for a set of ligands follows the Irving-Williams series [107], which ranks the metal ion complexes based on their stability in the following order:

$${\text{Mg}}^{2+}<{\text{Mn}}^{2+}<{\text{Fe}}^{2+}<{\text{Co}}^{2+}<{\text{Ni}}^{2+}<{\text{Cu}}^{2+}>{\text{Zn}}^{2+}$$

This signifies that metal ions such as Zn^2+^ or Cu^2+^ form the most stable complexes with a particular set of ligands. However, by increasing the total concentration of a weaker binding metal cation, the cell can enhance its competitiveness for a target protein if the buffering allows for an increased availability.

The cytosolic free concentrations of essential metal ions vary over many orders of magnitude and they are kept in defined ranges by buffering in order to avoid overlapping functions of the different metal ions (Table 6). Although the concentration of some metal ions is low at steady state, there are instances where it increases either globally or locally. For instance, the cytosolic Ca^2+^ concentration increases in response to second messengers such as inositol trisphosphate, IP_3_ [108]. Zinc ion concentrations also increase in response to various stimuli such as high glucose concentrations, insulin, growth factors, and changes to a lower reducing potential [109], [110], [111].

#### 3.1.1 Zinc(II)

Zinc ions have been referred to as insulin-mimetics as incubation of cells with zinc ions results in glucose transport [120] and lipogenesis [121]. The insulin-mimetic effects are associated with an increased tyrosine phosphorylation, and it has been suggested that these effects are due to a direct interaction of zinc ions with PTPs [35]. There is a large body of evidence suggesting that zinc ions are indeed *bona fide* endogenous modulators of PTPs, putting the observation of zinc ion inhibition observed *in vitro* in a physiological context. Some PTPs such as PTPRB could be inhibited at resting zinc ion concentrations [26], while others will need increased cytosolic zinc ion concentrations as the inhibition constants are above the cytosolic free zinc ion concentrations (Table 3).

**Fig. 8:**
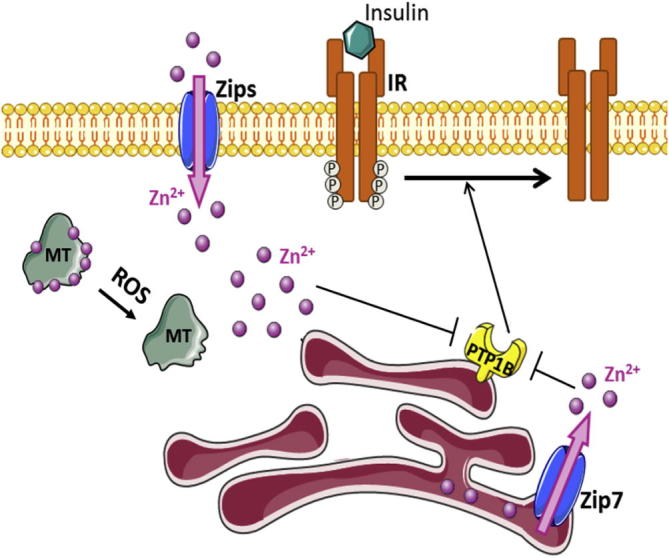
Mechanisms involved in increasing cytosolic zinc after insulin stimulation. Insulin binding to its receptor induces zinc increase through the zinc transporter Zip7 located on the endoplasmic reticulum (ER) membrane or through other Zips located on the plasma membrane. Insulin stimulates the insulin receptor (IR), which triggers an increase in ROS production. Oxidation of metallothioneins (MT) releases the bound zinc ions. Zinc then inhibits PTP1B, hence blocking the dephosphorylation of the insulin receptor. The figure was made using Servier Medical Art (www.servier.com).

Cellular zinc ion homeostasis is tightly controlled. It is fairly well established now that the concentration of free zinc ions in the cytosol is in the range of a few hundred pM [112], [113]. The concentration of zinc ions inside intracellular organelles, however, is more controversial and investigations using different fluorescent probes provided very different values [122], [123]. Metallothionein is a major zinc ion-binding protein contributing to maintaining the cytosolic free zinc ion concentrations at high picomolar/low nanomolar concentrations [112], [124]. Two families of zinc transporters also participate in this control. Fourteen members of importers (Zip/*SLC39A*) and at least nine out of the ten members of transporters (ZnT/*SLC30A*) transport zinc ions into or out of the cytosol [125]. These proteins are located on the plasma membrane as well as on membranes of intracellular organelles. The release of zinc ions into the cytosol through some of these transporters results in free zinc ion concentrations that are locally higher than those under steady-state conditions. Stimuli such as prolonged hyperglycemia [109], EGF [111] or insulin [110] increase the cellular free zinc ion concentration. Two pathways may be relevant for local increases of zinc ions at the endoplasmic reticulum (ER) where PTP1B resides. One pathway is the transport of zinc ions through zinc transporters (Fig. 8). Zip7 (*SLC39A7*) is located on the ER membrane and responsible for transporting zinc ions into the cytosol. Stimulation of cells with EGF in the presence of high intracellular Ca^2+^ activates casein kinase 2 (CK2) that is consequently recruited to the ER, where it phosphorylates Zip7 on Ser^275^ and Ser^276^ [111]. This modification causes an opening of the channel and increases in zinc ion concentrations in the cytosol. However, because zinc ions are buffered in the cytosol, the global free zinc concentration would not be expected to change. The concentration of zinc at the ER membrane would be high enough for PTP1B inhibition. Insulin increases cellular free zinc ion concentrations. For example, treatment of a T-cell lymphoma cell line with 5–10 μg/mL insulin elicits a 2–3.5-fold increase of zinc ion concentrations [110]. To rule out that the increase of intracellular zinc ions in these experiments was due to an influx of zinc contained in the insulin preparation, which often contains rather high concentrations of zinc ions, the same experiment was repeated after boiling the insulin sample and hence denaturing the hormone. No increase in fluorescence was observed with this preparation [110].

Increases of cellular free zinc ions can also arise from the production of reactive oxygen species (ROS) and oxidation of metallothioneins (MTs) (Fig. 8). Insulin binding and activation of the insulin receptor induces an increase of intracellular H_2_O_2_ in both hepatocytes and adipocytes [126]. ROS are produced from Nox4, the phagocytic NAD(P)H oxidase catalytic subunit, which is highly expressed in insulin-sensitive adipocytes. Expression of dominant negative forms attenuated insulin-stimulated H_2_O_2_ production in adipocytes while silencing of Nox4 inhibited the insulin signaling cascade [127]. Targets of H_2_O_2_ are PTPs, in particular PTP1B, which is reversibly inhibited, thus enhancing insulin signaling pathways [128] (Section 3.2). A second mode of action is the reaction of H_2_O_2_ with MTs. Oxidation of MTs results in release of tightly bound zinc ions, which also inhibit PTP1B [129], [130]. Insulin stimulation therefore enhances its own signal through inhibition of PTP1B by redox and zinc ion signals (Fig. 8). Incubation of adipocytes in a medium containing high glucose concentrations resulted in enhanced H_2_O_2_ production and PTP oxidation [131]. Incubation of cells with high glucose concentrations also has been linked with an increase in intracellular free zinc ion concentrations [109], demonstrating the joint action of redox and zinc signals on modulation of PTP activity. However, the mode of inhibition by zinc ions suggests that modulation of PTP1B activity by either oxidation or zinc ions occurs at two distinct stages. PTP1B is oxidized at the resting level, independently of physiological stimulation and substrate binding. In contrast, zinc ions modulate PTP1B after physiological stimulation and substrate binding. Aside from the fact that zinc ion signals can be a product of oxidative signaling, zinc ion signals are specific based on affinities of zinc ions for coordination environments and can provide a longer lasting inhibition of PTPs [132].

#### 3.1.2 Other metal ions

The inhibition of PTP1B by metal ions other than zinc ions does not appear to be physiologically significant but it may have significance under conditions where tissues and cells are exposed to toxic metal ion concentrations [37].

### 3.2 Redox regulation of PTP1B

**Fig. 9:**
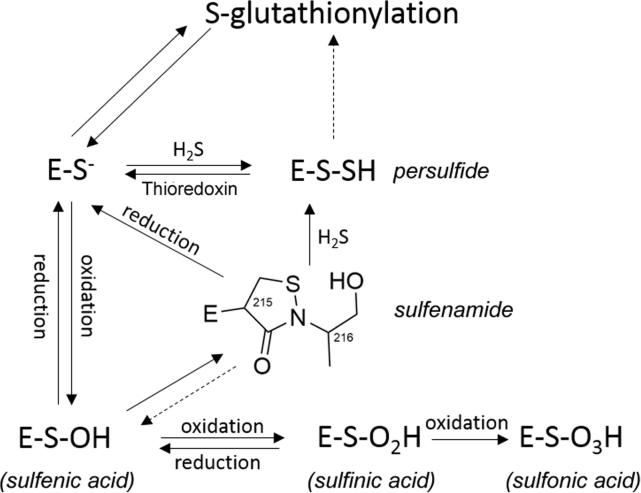
Redox chemistry of the active site cysteine in PTPs.

Oxidation/reduction (redox) is a key regulatory event in PTP function. Intracellular signals resulting in the production of ROS increase tyrosine phosphorylation due to inhibition of PTPs [133]. ROS oxidize the active site cysteine (Cys^215^ in PTP1B) and abrogate its nucleophilic function. Oxidation is reversible as the sulfenic acid form of the oxidized cysteine reacts with the adjacent serine side chain (Ser^216^) to form a cyclic sulfenamide with the purpose of avoiding further oxidation and facilitating reduction to restore the active PTP [134] (Fig. 9). Formation of the cyclic sulfenamide is accompanied by conformational changes as the PTP loop with the signature motif VHCxAGxxRSG and Tyr^46^ (in PTP1B), normally buried in the structure, become more solvent-exposed.

Oxidation can also lead to the formation of sulfinic or sulfonic acid derivatives of Cys^215^ [7], [134]. The oxidation to sulfonic acid is irreversible. Additional modifications are glutathionylation, sulfenation with hydrogen sulfide and reactions with reactive selenium compounds. The reduction of the oxidized cysteine depends on an interaction of the protein with thioredoxin [135], [136].

### 3.3 PTP1B as a drug target

Given the role of PTP1B in the pathogenesis of type 2 diabetes and its involvement in cancer signaling, immense research efforts are made to pharmacologically targeting the enzyme [137]. Antisense-based oligonucleotides designed to downregulate PTP1B expression and normalizing glycaemia and improving insulin sensitivity have entered Phase II clinical trials [138], [139]. Although the trial is over, the results are not yet available to the public [140].

There are two main challenges when designing PTP inhibitors: cell membrane permeability and selectivity [137]. Inhibitors for PTPs, and in particular PTP1B, include phosphate mimetics, such as vanadium compounds. Chelation overcomes the issues of cell membrane permeability, and a barrier of positive charges around the active site. Selectivity continues to be an issue as many PTPs share common residues in their catalytic sites.

Since diabetes and other diseases are associated with changes in zinc status, one would assume that an approach targeting intracellular control of zinc is worth exploring for pharmacological intervention.

## 4 Conclusions

Phosphatases are the jockeys while kinases are the racehorses in cellular regulation. Phosphatases perform the task of *regulating* phosphorylation signaling and they are regulated themselves. We demonstrate additional principles of the regulation of PTPs, namely reversible inhibition by metal cations that inhibit differently from metal anions such as vanadate. Oxyanions bind to the enzymes in the open conformation but cations inhibit only if an oxyanion such as phosphate (or vanadate) is already present and new interactions for cation binding are made possible in the closed conformation of the protein when the catalytic aspartate moves into the active site.

Metal cation inhibition modulates PTPs differently from established redox modulation. Metal cations bind when phosphate (or another oxyanion) blocks the cysteine in the phospho-intermediate, and thus the sulfur is no longer available for redox modification. Possibly, the combined anion/cation inhibition protects the enzyme from redox modification.

Metal modulation has been ignored in virtually all discussions of the biology of PTPs. The inhibition of PTPs by metal ions was recognized when these enzymes were initially characterized [26]. The reasons for a lack of progress are that the structural chemistry of metal cation inhibition was not explored, and in fact could not have been explored, because it requires a particular intermediate of the enzyme in the reaction cycle, and that the relationship between physiological zinc ion concentrations and inhibition was not recognized. Oxyanion modulation alone is not important physiologically unless it is seen in the context of metal cation binding. There is controlled zinc ion release at the ER where PTP1B resides and it is now understood that physiological concentrations of zinc ions are commensurate with the inhibition constants of PTPs for zinc ions.

**Fig. 10:**
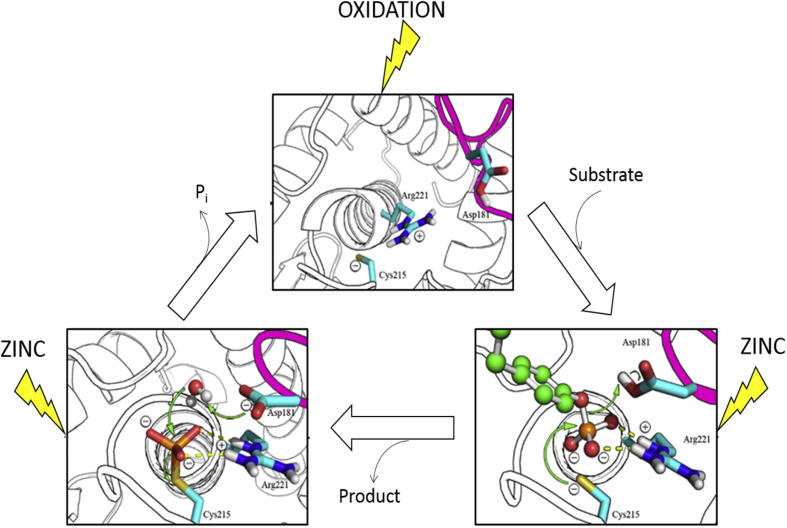
Modulation of PTP1B by oxidation of the catalytic cysteine and zinc binding at different stages of the catalytic cycle. While oxidation targets the enzyme in the open conformation, zinc modulates the enzyme after the WPD loop has closed. Modified from Brandao et al. [24].

Transition metal ions are redox active and influence the redox state of the catalytic cysteine in PTPs. In contrast, zinc(II) is redox-inert in biology and cannot change the redox state of the cysteine. However, ROS release zinc ions from metallothionein and other protein sites and thus increase free zinc ion concentrations. Therefore, some of the effects of ROS on PTPs may be indirect, namely in providing zinc ions as a specific inhibitor of the enzymes. In as much as redox species are ER localized, so is zinc ion release, and specific pathways of zinc ion release support the physiological relevance. The physiological context of dual modulation of PTPs by metal ions and redox is likely another level of control of these important enzymes (Fig. 10).

The interplay of anions, cations and redox agents provides a very rich chemistry for affecting the active site of PTPs and consequently their biological control. On a more speculative note, the zinc ion inhibition of the phosphorylated form relates the activity of the PTPs to the phosphorylation state of their substrates, *i.e.* inhibition occurs during enzymatic turnover while redox modulation targets the free enzyme before (or after) it has been enzymatically active.

Metal ion interactions with PTPs extend the role of metal ions in phosphatase action and phosphorylation signaling. Alkaline phosphatases use a binuclear zinc site and a regulatory magnesium ion, acid phosphatases a binuclear iron site, and protein phosphatases such as calcineurin a mixed binuclear zinc/iron site. Thus, in addition to using metalloenzymes in the control of phosphorylation signaling, biology has availed itself to using metal ions such as zinc and calcium for biological regulation of phosphatases.

It is remarkable that billions of dollars have been spent on drug design and clinical trials of inhibitors of PTPs without appreciating these structural and biological principles of regulation. The new insights provide a different perspective and may give another impetus for rational drug design and for inhibiting PTPs more specifically by modulating zinc metabolism *in vivo*. Such an approach appears to be attractive since the zinc and vanadium inhibition of PTP1B provides a mechanistic basis for the rather copious literature on the pharmacology of insulin-mimetic and anti-diabetic effects of zinc and vanadium complexes [141], [142], [143].

## Figures and Tables

**Table 1:** Free zinc ion concentrations calculated using MaxChelator and MINEQL+ 4.6 software and compared to the total zinc ion concentration. The buffer composition (50 mM Hepes/Na^+^, 0.1 mM tris-carboxyethylphosphine (TCEP), and 1 mM nitrilotriacetic acid (NTA)), pH (7.4), temperature (25 °C), and ionic strength were taken into account to calculate the free zinc ion concentrations [28].

Zn^2+^ (Tot) (μM)	[Zn^2+^]_free_ (M) MaxChelator	[Zn^2+^]_free_ (M) MINEQL+
10	2.66 × 10^−11^	2.93 × 10^−11^
30	8.14 × 10^−11^	8.98 × 10^−11^
100	2.93 × 10^−10^	3.22 × 10^−10^
300	1.13 × 10^−9^	1.24 × 10^−9^
500	2.63 × 10^−9^	2.90 × 10^−9^
600	3.95 × 10^−9^	4.34 × 10^−9^
700	6.15 × 10^−9^	6.72 × 10^−9^
800	1.05 × 10^−8^	1.14 × 10^−8^
900	2.37 × 10^−8^	2.45 × 10^−8^
950	5.01 × 10^−8^	4.45 × 10^−8^
990	2.56 × 10^−7^	8.87 × 10^−8^
1000	1.70 × 10^−6^	1.06 × 10^−7^

**Table 2:** Amino acids in the metal ion-binding sites of proteins. Frequencies of each amino acid in a given type of metal ion-binding site are tabulated; where not shown the frequency is below 4%. Metal ion-binding polypeptides were extracted from the RCSB Protein Data Bank (PDB).

Amino acid	Metal ion						
	Ca^2+^	Cd^2+^	Cu^2+^	Fe^3+^	Mg^2+^	Mn^2+^	Zn^2+^
Arg	–	–	–	–	–	–	–
Asn	10–15%^a^	–	–	–	5–10%^a^	∼5%^a^	–
Asp	35–40%^a^ 36%^b^	24%^b^	∼5%^a^	10–15%^a^	35–40%^a^ 35%^b^	40–45%^a^ 37%^b^	10–15%^a^ 17%^b^
Cys	–	10%^b^	10–15%^a^	10–15%^a^	–	–	40–45%^a^ 23%^b^
Glu	15–20%^a^ 27%^b^	27%^b^	∼5%^a^	–	15–20%^a^ 22%^b^	15–20%^a^ 25%^b^	10–15%^a^ 15%^b^
Gln	–	–	–	–	–	–	–
Gly	5–10%^a^	–	–	–	–	–	–
His	–	26%^b^	65–70%^a^	45–50%^a^	∼5%^a^	20–25%^a^ 26%^b^	30–35%^a^ 36%^b^
Met	–	–	5–10%^a^	–	–	–	–
Ser	∼5%^a^	–	–	–	5–10%^a^	–	–
Thr	∼5%^a^	–	–	–	5–10%^a^	–	–
Tyr	–	–	∼5%^a^	5–10%^a^	–	–	–

a Metal ion-binding sites were defined as residues within 3.5 Å of the metal ion [30].

b Metal ion-binding sites were defined as residues within 2.5 Å of the metal ion [31].

**Table 3:** Zinc(II) inhibition of human protein tyrosine phosphatases.

Protein tyrosine phosphatase	Inhibition	Reference
PTP1B	IC_50_ = 3–17 nM	[28], [34]
SHP-1	IC_50_ = 93 nM	[35]
SHP-2	IC_50_ = 1–2 μM	[34]
LAR (PTPRF)	IC_50_ = 20 μM	[34]
TC-PTP	IC_50_ = 200 nM	[4]
PTPRB	IC_50_ = 98 pM; *K*_i_ = 21 pM	[26]
PTEN	IC_50_ = 0.6 nM	[39]

**Table 4:** Potential zinc ion-coordinating ligands in three structures of PTP1B as obtained from molecular docking [28].

PDB id	Pose 1^a^	Pose 2	Pose 3	Pose 4	Pose 5
2CM2	Lys141 Thr143 Glu159 Glu161	Glu2 Glu4 Glu6	Glu4 Lys5 Glu8	His0 His1 Glu2 Asp240	Asp29 Pro31 Cys32 Met258

3I80	Lys141 Thr143 Glu159 Glu161	Glu2 Met3 Glu4 Ile275 Ala278	Glu8 Asp11	Asp181 Cys215 Arg221 Gln262	Asp289 Lys292 Glu293

1A5Y	Met74 Glu75 Glu76 Asp252	His60 Glu101	Asp181 P-Cys215 Gln266	His25 Glu26	Glu129 Glu132

a Candidate binding modes are referred to as “poses” in molecular docking.

**Table 5:** Oxyanions in biological systems.

Name of ion	Formula^a^	Charge^a^	PDB code	# entries	Geometry^b^^,^^c^
Arsenate	HAsO_4_^2^^−^	−2	ART	3	TH
Chromate	H_2_CrO_4_^−^	−1	–	0	–
Molybdate	MoO_4_^2^^−^	−2	MOO	22	TH, TB
Phosphate	HPO_4_^2^^−^	−2	PO4	4156	TH
Sulfate	SO_4_^2^^−^	−2	SO4	14234	TH, TB
Tungstate	WO_4_^2^^−^	−2	WO4	48	TH, TB
Vanadate	H_2_VO_4_^−^	−1	VO4	80	TH, TB

a Observed under standard condition of temperature (25 °C) and pH (7.4).

b TH – tetrahedral; TB – trigonal bipyramidal.

c From the Protein Data Bank (PDB).

**Table 6:** Intracellular free metal ion concentrations ([Me^2+^]_i_).

Metal	Free [Me^2+^]_i_	Reference
Zn	600 pM–1 nM	[112], [113]
Fe	<5 μM	[114], [115]
Cu	1 aM	[116]
Ca	100 nM	[117]
Mg	1 mM	[118], [119]
